# Is sexual autonomy a protective factor for neonatal, child, and infant mortality? A multi-country analysis

**DOI:** 10.1371/journal.pone.0212413

**Published:** 2019-02-22

**Authors:** Peter Memiah, Yvonne Opanga, Tristi Bond, Courtney Cook, Michelle Mwangi, Jenna Fried, Marie A. Joseph, Kevin Owuor, Vernon Mochache, Yvonne Wangui Machira

**Affiliations:** 1 Department of Public Health, Usha Kundu College of Health, University of West Florida, Pensacola, Florida, United States of America; 2 Amref Health Africa, Nairobi, Kenya; 3 Biology Department, University of West Florida, Pensacola, Florida, United States of America; 4 Department of Economics, University of Nairobi, Nairobi, Kenya; 5 Psychology Department, University of West Florida, Pensacola, Florida, United States of America; 6 Centre for Health Solutions, Nairobi, Kenya; 7 Kenya National AIDS Control Council, Nairobi, Kenya; 8 Tafiti Research Group, Nairobi, Kenya; University of Botswana, BOTSWANA

## Abstract

**Background:**

Sexual autonomy empowers women to set boundaries, take control of their bodies, prevent sexually transmitted diseases and avoid unplanned pregnancy. A woman’s ability to negotiate safer sex is crucial for her survival and that of her child. Sexual autonomy among East African women is vital to the elimination of the deaths of neonates, infants, and children. The aim of our study was to explore the association of sexual autonomy on neonatal, infant, and child mortality.

**Methodology:**

This was a secondary analysis of demographic health survey (DHS) data on women of reproductive age (15–49 years) in five East African countries: Burundi, Kenya, Rwanda, Tanzania, and Uganda. Data on our outcome variables neonatal, infant, and under-five mortality which were in binary form was extracted from the database. Sexual autonomy was classified as a composite variable of “respondent can refuse sex,” “respondent can ask partner to use condom,” and “if spouse is justified in asking husband to use condom.” Other sociodemographic, maternal, health system and paternal variables were included in the analysis. STATA version 14 was used for analysis. Proportions and frequencies were used to describe the three outcome variables and sociodemographic characteristics. Chi-square tests were used to compare associations between sexual autonomy and categorical variables. Adjusted hazard ratios were used to determine the association between sexual autonomy and independent variables.

**Results:**

The sampled women were predominantly urban (75%; n = 5758) and poor (48.7%; n = 3702). A majority of those that experienced mortality (neonatal mortality 53.5%, infant mortality 54.3%, under-five mortality 55.7%) were young (under 20) at the time of their first child’s birth while their male partners were older. The multivariate analysis supports the beneficial effects of women’s sexual autonomy in East Africa. Women who exercised sexual autonomy experienced significantly lower rates of child mortality at all three stages: neonatal (NHR = 0.80, 95% CI: 0.68–0.94, *p* = 0.006), infant (IHR = 0.82, 95% CI: 0.72–0.93, *p* = 0.003), and under-five (UHR = 0.84, 95% CI: 0.75–0.94, *p* = 0.002), net of all other factors. Receiving antenatal care and using contraceptives also contributed significantly to lower child mortality rates.

**Conclusion:**

Our findings suggest that sexual autonomy among East African women is an urgent priority that is crucial to the survival of neonates, infants, and children in East Africa. Women should be informed, empowered, and autonomous concerning their reproductive and sexual health.

## Introduction

Sexual autonomy among women is described as the ability to refuse sex or request that the partner uses contraception, such as a condom [1]. By doing so, women are setting boundaries, taking control of their own bodies, and working towards the prevention of sexually transmitted disease and unexpected pregnancy. Studies have shown that demographic characteristics of both mother and child can have an impact on neonatal, infant, and child mortality [2]. Sexual autonomy is conceptualized as a human right to protect and maintain an informed decision over one's body, one's sexuality, and one's sexual experience. Traditional gender roles include expectations for men to initiate sexual activity and for women to respond to men's attempts to initiate sexual behavior [3]. By testing gender and social norms, environments enabling sexual and reproductive health can show promise in reducing mortality rates among neonates, infants, and children [4].

The ability of a mother to fulfill her role in caring for her children can be reduced because of disruptions that occur due to lack of education, a poor understanding of sexual autonomy, and loss of control especially in a marital context. Without ways to boost autonomy and empowerment, these mothers find themselves at a greater risk of losing their children [5]. To prevent mortality, a woman’s ability to negotiate safer sex is crucial in sub-Saharan Africa [6]. For an environment to provide and promote sexual empowerment to women, peer support and male involvement can increase awareness of this public health issue while creating laws that will protect the fundamental human rights of all [4].

Without sexual autonomy, women are at risk of unwanted pregnancy, STDs, sexual coercion, and violence [7]. A woman’s ability to engage in safe sexual practices is dependent on her partner's willingness to take measures to avoid unintended pregnancy and STDs. It is logical to assume that women who are less aware of sexual rights have a difficult time refusing unwanted sexual advances and may be targeted by aggressive men [8].

An exploration of the association of sexual autonomy on neonatal, infant, and child survival was undertaken by using data collected from a nationally representative sample of women 15–49 years of age within the East African countries of Burundi, Kenya, Rwanda, Tanzania, and Uganda.

## Methods

A secondary analysis of the Demographic Health Survey (DHS) data on Women in East Africa was done from the East African countries of Burundi (DHS conducted in 2016–2017), Kenya (2014), Tanzania (2017), Uganda (2016), and Rwanda (2014–2015). The analysis is based on data collected from women of reproductive age (15–49 years).

### Study design

The Demographic Health Survey is a household–based, nationally representative cross sectional study conducted by ICF Macro/MEASURE DHS on behalf of national Ministries of Health with financial support from the United States Agency for International Development [9]. DHS data has previously been used in cross-country analyses in several studies. DHS surveys are nationally representative, population-based household surveys that employ standardized questionnaires and modules for household, women’s, and men’s interviews.

### Sample design

Nationally representative of households were selected in the five East African countries (36430 Kenya, 12563 Tanzania, 19588 Uganda, 12699 Rwanda, and 8596 Burundi). The total number of women from each country, who were of reproductive age 15–49, included in our sample based on the response to our outcome variable is as follows: Kenya, 2,432; Tanzania, 2001; Uganda, 3579; Rwanda, 1479; and Burundi, 3031.

DHS used a multistage, clustered area sampling technique. In the first sampling stage, each country was stratified into major sub-national regions from which census-based enumeration areas were selected with probability proportional to size. The major regions may or may not coincide with administrative units (as in Uganda) and consist of provinces or groups of provinces. The Kenya 2014 and Rwanda 2014–15 surveys stratified districts in the first sampling stage. Urban areas and less populous areas were typically oversampled in the first sampling stage in order to produce reliable regional estimates and rural-urban comparisons of health indicators. A mapping and household listing exercise was then implemented in each selected enumeration area. In the second sampling stage, households were randomly selected from the household list within each enumeration area. Table 1 lists the countries included in our analysis and the corresponding survey years.

**Table 1:** List of East African countries included in analysis.

Country	Year	Population (no.)	GDP per Capita (USD)	Crude Birth Rate per 1000	Maternal Mortality per 100,000 Live Births	Under-5 Mortality per 1,000 Live Births	Neonatal Mortality per 1,000 Live Births	Sexual Violence 15–49 Years	Condom Use Among Women 15–49 Years
Burundi	2015–2016	10.8 M	285.7	34	334	71.7	24.2	23%	1.0%
Kenya	2014–2015	43 M	1143.1	30.5	362	52	22	14%	3.1%
Rwanda	2015–2016	10.5 M	719	32.6	210	50	20	22.4%	2.2%
Tanzania	2015–2016	50.1 M	867	37.2	398	67	25	17%	3.9%
Uganda	2015–2016	41 M	580.4	42.1	432	64	27	39%	1.9%

### Conceptual framework and study variables

A literature search using PubMed, Google Scholar, and HINARI was conducted on the relationship between sexual autonomy and neonatal, infant, and child survival in Sub-Saharan Africa. Among the identified variables were three outcome variables including: 1) Neonatal mortality (NM) as reported by the mothers who participated in the survey; it was defined as the death of a neonate between birth and 1 month of life, 2) Infant mortality (IM) was defined as the death of an infant before his/her first birthday, and 3) Child Mortality, also known as under-five mortality (UM), refers to the death of infants and children under the age of five. All the variables took a binary form, such that neonatal death will be regarded as a success (1  =  if death occurs in the specified age period) or failure (0  =  if the newborn/infant/child is alive in the specified age period). The outcome variable was examined against sexual autonomy and then against all the confounding variables as indicated.

**Fig 1:**
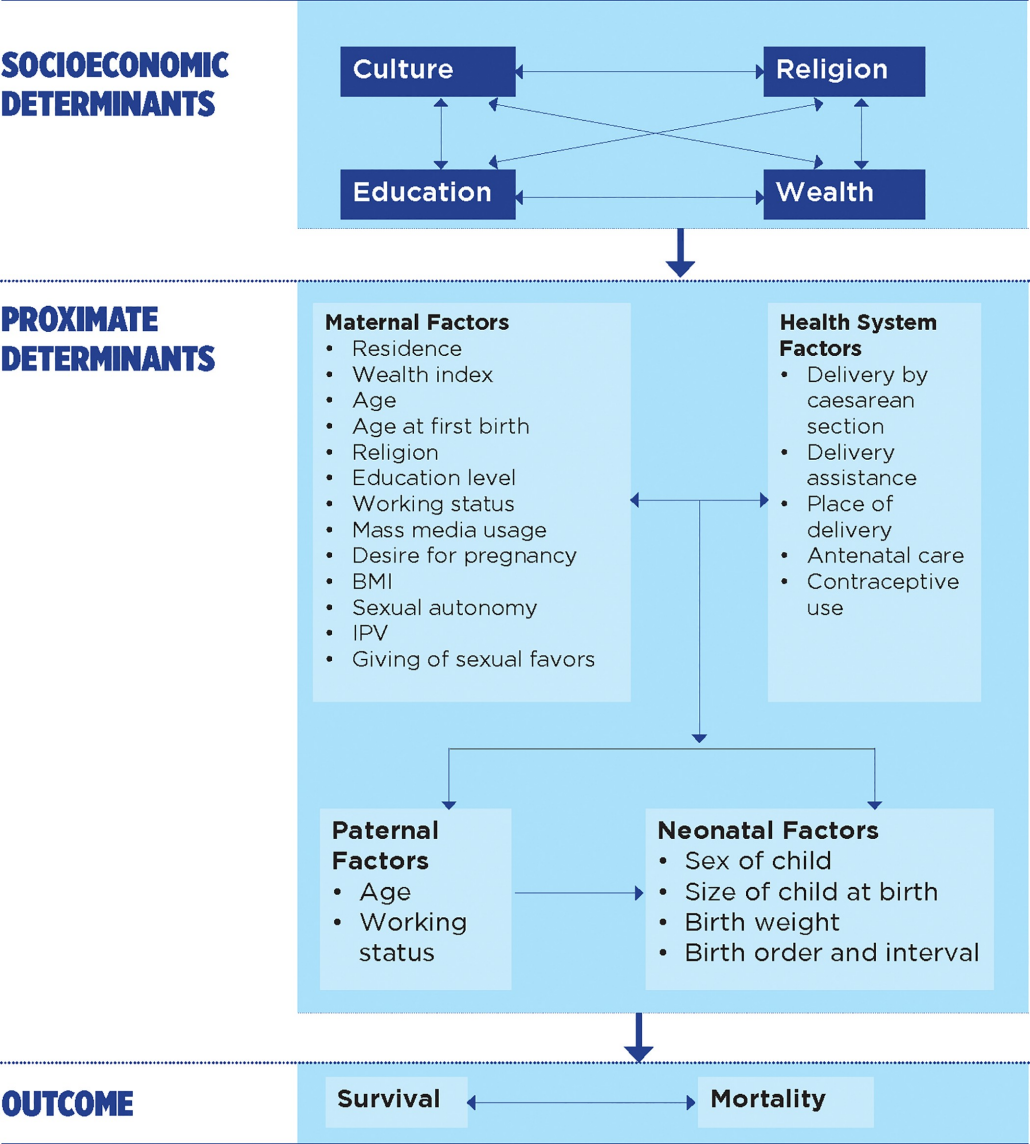
Conceptual framework.

The study adopted a conceptual framework developed by Mosley and Chen in 1984. The framework combines sociological and biological variables into distal and proximate determinants of neonatal, child, and infant mortality. This framework has been used extensively in other similar studies in understanding child survival [10–12]. The framework utilizes a systematic approach which addresses the effects of the factors in the previous level. The distal variables are socioeconomic determinants and community factors while the proximate factors are health status factors of both mother and neonate, infant, or child, as indicated in Fig 1 and Table 2.

**Table 2:** Variable description and categorization.

Variables	Description and Categorization
**Community Level Factors**	
Residence	Area of residence (1 = Rural; 2 = Urban)
**Socio-economic Factors**	
Wealth index	Household wealth index (1 = Poor; 2 = Middle; 3 = Rich)
Mother’s age	Age of mother (1 = <20; 2 = 20–29; 3 = 30–49)
Mother’s age at first birth	Age of mother at first birth (1 = <20; 2 = 20–29, 3 = 30–49)
Husband/partner’s age	Age of husband (1 = <29; 2 = 30–39; 3 = 40–49; 4 = 50 plus)
Mother’s religion	Mother’s religion (1 = Roman catholic; 2 = Protestant/Other Christian; 3 = Muslim; 4 = No religion)
Mother’s education level	Mother’s education level (1 = No education; 2 = Primary; 3 = Secondary; 4 = Higher)
Mother’s working status	Mother’s working status (1 = No; 2 = Yes)
Father’s working status	Father’s working status (1 = No; 2 = Yes)
Mother’s mass media usage	Mother’s mass media usage (1 = Yes; 2 = No)
**Health-related Factors**	
Desire for pregnancy	Mother’s desire to have a baby (1 = Then; 2 = Later; 3 = No more)
BMI (kg/m^2^)	Mother’s body mass index (1 = <18.5; 2 = > = 18.5)
Sex of child	Sex of child (1 = Male; 2 = Female)
Size of child at birth	Size of child at birth (1 = Average or larger; 2 = Small or very small; 3 = Missing)
Birth weight (g)	Weight of child at birth (1 = <2500; 2 = 2500–3500; 3 = >3500; 4 = Not weighed; 5 = Don’t know)
Birth order and interval	Birth order and birth interval (1 = 2^nd^/3^rd^ child, >2 years; 2 = 1^st^ child; 3 = 2^nd^/3^rd^ child, < = 2 years; 4 = 4^th^/higher child, >2 years; 5 = 4^th^/higher child, < = 2 years)
Delivery by caesarean section	Delivery by caesarean section (1 = No; 2 = Yes)
Delivery assistance	Professional assistance during birth (1 = Non-health professional; 2 = Health professional)
Place of delivery	Place of delivery (1 = Home delivery; 2 = Hospital/Other)
Antenatal care	Antenatal care received by mother during pregnancy (1 = No; 2 = Yes)
Sexual autonomy	Mother’s consent to engage in sexual activities (1 = No; 2 = Yes)
Mother experienced IPV	Mother experienced IPV (1 = No; 2 = Yes)
Contraceptive use	Mother’s contraceptive use (1 = No; 2 = Yes)
Mother giving sexual favors	Mother seeking to provide sexual favors for economic gain (1 = No; 2 = Yes)

The **variable of interest** was sexual autonomy which was classified as a composite variable of “respondent can refuse sex,” “respondent can ask partner to use condom,” and “wife is justified in asking the husband to use condom.” Other key variables included sociodemographic variables, such as age, marital status, educational level, religion, and place of residence (rural or urban). Additionally, we included data on income-related variables like occupational status and wealth Index as well as sexual and behavioral variables, including sexual debut, number of sexual partners, and whether the individual received money, gifts, or favors in return for sex. Partner-related variables included partner age and occupational status as indicated in Table 2.

### Data abstraction and analysis

The survey data was downloaded from each of the five countries’ DHS websites after permission to utilize the data was granted. The data was then cleaned and exported to STATA version 14 for analysis. Univariate, bivariate, and multivariate analyses were conducted. Frequencies and proportions were first conducted and then cross tabulations to compare the proportions of outcome variables and sexual behavioral variables across each of the countries. At the bivariate level, chi-square tests were used to determine the association between sexual autonomy and categorical variables. The associations between the sexual autonomy and each of the independent variables were measured by means of odds ratios at 95% confidence intervals. Variables of significance at the bivariate level of analysis were further analyzed at the multivariate level using logistic regression. Adjusted odds ratios were used to determine the association between sexual autonomy and independent variables. Sampling weights included in the datasets were applied. These weights account for both sampling probability and non-response. In this study, the weight for sample selection and non-response from our data was utilized. In addition, the complex survey (svy) commands available within STATA 14 accounted for clustered sampling design and estimated robust standard errors which was the basis for the 95% confidence intervals reported in the following sections.

## Results

### Neonatal, infant and under-five mortality rates in East Africa

As noted above in Table 1, the sample used in this analysis includes women of reproductive age (15–49) in all five countries of the East African region. Table 3 presents basic demographic characteristics of the sampled women for each of the three child mortality outcome measures (NM = neonatal mortality, IM = infant mortality, UM = under-five mortality). The sampled women were predominantly urban and poor. A majority (NM 53.5%, IM 54.3%, UM 55.7%) were young (under 20) at the time of their first child’s birth while their male partners were older. About a quarter (NM 23.3%, IM 24.9%, UM 26.4%) of the women had no education, but a large majority were working (NM 78.0%, IM 79.3%, UM 79.1%). Employment rates among women were still less than that of men. About two-thirds of the women (NM 68.7%, IM 69.2%, UM 69.6%) were ready to have another child, and only about 8 percent did not desire more children. Less than a third (NM 31.1%, IM 30.4%, UM 29.3%) were using contraceptives. A large majority of women received antenatal care (NM 91.7%, IM 92.2%, UM 92.7%), gave birth in a hospital (NM 71.1%, IM 70.4%, UM 69.7%) and were attended to by healthcare professionals (NM 69.7%, IM 69.9%, UM 70.1%)—all likely due to their predominantly urban residence. The majority of women (NM 57.8%, IM 57.5%, UM 57.9%) reported experiencing some form of intimate partner violence (IPV).

**Table 3:** Characteristics of respondents used in the analysis of neonatal, infant, and under-five mortality in East Africa.

Variable	East Africa (Kenya, Uganda, Tanzania, Rwanda, Burundi)		
	Neonatal Mortality	Infant Mortality	Under-five Mortality
**Residential Area**			
Rural	423 (24.5)	610 (23.7)	729 (22.6)
Urban	1306 (75.5)	1960 (76.3)	2492 (77.4)
**Wealth Index**			
Poor	797 (46.1)	1258 (48.9)	1647 (51.1)
Middle	319 (18.4)	463 (18)	571 (17.7)
Rich	613 (35.5)	849 (33)	1003 (31.1)
**Mother’s Age**			
<20 years	110 (6.4)	144 (5.6)	164 (5.1)
20–29 years	834 (48.2)	1247 (48.5)	1584 (49.2)
30–49 years	785 (45.4)	1179 (45.9)	1473 (45.7)
**Mother’s Age at First Birth**			
<20 years	925 (53.5)	1395 (54.3)	1795 (55.7)
20–29 years	778 (45)	1135 (44.2)	1378 (42.8)
30–49 years	26 (1.5)	40 (1.6)	48 (1.5)
**Husband/Partner’s Age**			
<29 years	348 (28.3)	482 (26.5)	620 (26.8)
30–39 years	467 (38)	737 (40.5)	926 (40.1)
40–49 years	297 (24.2)	427 (23.5)	533 (23.1)
50 plus years	116 (9.4)	172 (9.5)	232 (10)
**Mother’s Religion**			
Roman Catholic	505 (34.9)	750 (34.8)	936 (34.7)
Protestant/Other Christian	657 (45.4)	989 (45.8)	1233 (45.7)
Muslim	194 (13.4)	269 (12.5)	327 (12.1)
No religion	92 (6.4)	150 (7)	204 (7.6)
**Mother’s Education Level**			
No education	403 (23.3)	641 (24.9)	850 (26.4)
Primary	1002 (58)	1485 (57.8)	1866 (57.9)
Secondary	277 (16)	376 (14.6)	432 (13.4)
Higher	47 (2.7)	68 (2.6)	73 (2.3)
**Mother’s Working Status**			
No	325 (22)	454 (20.7)	577 (20.9)
Yes	1154 (78)	1735 (79.3)	2187 (79.1)
**Father’s Working Status**			
No	34 (2.7)	55 (2.9)	73 (3.1)
Yes	1226 (97.3)	1824 (97.1)	2314 (96.9)
**Mother’s Mass Media Usage**			
Yes	469 (27.1)	728 (28.3)	960 (29.8)
No	1260 (72.9)	1842 (71.7)	2261 (70.2)
**Desire for Pregnancy**			
Then	1010 (68.7)	1507 (69.2)	1914 (69.6)
Later	341 (23.2)	501 (23)	631 (22.9)
No more	120 (8.2)	171 (7.8)	206 (7.5)
**Mother’s BMI**			
<18.5	68 (7.1)	111 (7.9)	144 (8.2)
> = 18.5	888 (92.9)	1289 (92.1)	1616 (91.8)
**Sex of Child**			
Male	988 (57.1)	1454 (56.6)	1793 (55.7)
Female	741 (42.9)	1116 (43.4)	1428 (44.3)
**Size of Child at Birth**			
Average or larger	960 (65.3)	1523 (69.9)	1989 (72.3)
Small or very small	446 (30.3)	586 (26.9)	682 (24.8)
Don’t know	64 (4.4)	69 (3.2)	79 (2.9)
**Birth Weight (g)**			
<2500	221 (15)	290 (13.3)	328 (11.9)
2500–3500	404 (27.5)	711 (32.7)	930 (33.8)
>3500	200 (13.6)	327 (15)	453 (16.5)
Not weighed	557 (37.9)	754 (34.6)	935 (34)
Don’t know	87 (5.9)	95 (4.4)	103 (3.7)
**Birth Order and Interval**			
2^nd^/3^rd^ child, >2 years	361 (20.9)	563 (21.9)	724 (22.5)
1^st^ child	463 (26.8)	633 (24.6)	778 (24.2)
2^nd^/3^rd^ child, ≤2 years	154 (8.9)	234 (9.1)	296 (9.2)
4^th^/higher child, >2 years	490 (28.3)	747 (29.1)	944 (29.3)
4^th^/higher child, ≤2 years	261 (15.1)	393 (15.3)	479 (14.9)
**Delivery by Caesarean Section**			
No	1555 (90.1)	2340 (91.4)	2956 (92.1)
Yes	170 (9.9)	220 (8.6)	254 (7.9)
**Delivery Assistance**			
Non-health professional	491 (30.3)	721 (30.1)	899 (29.9)
Health professional	1127 (69.7)	1676 (69.9)	2109 (70.1)
**Place of Delivery**			
Home delivery	493 (28.9)	751 (29.6)	964 (30.3)
Hospital/other	1215 (71.1)	1789 (70.4)	2221 (69.7)
**Antenatal Care**			
No	58 (8.3)	80 (7.8)	91 (7.3)
Yes	642 (91.7)	950 (92.2)	1154 (92.7)
**Mother Experienced IPV**			
No	624 (42.2)	930 (42.5)	1164 (42.1)
Yes	855 (57.8)	1260 (57.5)	1601 (57.9)
**Contraceptive Use**			
Not using	1192 (68.9)	1789 (69.6)	2276 (70.7)
Using	537 (31.1)	781 (30.4)	945 (29.3)
**Mother Giving Sexual Favors**			
No	212 (96.4)	311 (96.3)	369 (96.3)
Yes	8 (3.6)	12 (3.7)	14 (3.7)

Each cell represents the number and percentage in each category among sampled women of reproductive age (15–49).

Table 4 presents the estimated rates (per 1000) of three different measures of child mortality for the five countries combined, broken out by the demographic characteristics of the sampled women listed in Table 3. The rates are presented with 95% probability confidence intervals, and in most cases, the estimated rates for categories of a given demographic variable fall within the confidence intervals of the other categories, indicating no significant difference. This section highlights significant differences between categories of demographic variables. Estimated mortality rates were lower for women residing in urban areas than those in rural areas, but only for neonatal mortality did the difference approach significance (NMR: urban 0.25, rural 0.30). It appears that women aged 30–49 at their child’s first birth experienced higher rates on all three mortality measures. Due to the small number of women in this category, the confidence intervals were very wide and the differences were, therefore, not statistically significant. It is also notable that estimated rates of under-five mortality were as high for women who gave birth when under the age of 20 (UMR: 0.51, 95% CI: 0.48–0.54) as they were for the older women (UMR: 0.55, 95% CI: 0.38–0.79), likely indicating that factors in the child’s living environment during the early years play an important role after birth. Mothers with a BMI lower than 18.5 experienced neonatal mortality at lower rates (NMR: 0.17 vs. 0.28). The estimated rates of infant and under-five mortality for these women were also lower, but confidence ranges overlap. The child’s birth weight was a significant factor in child mortality at all three stages with small or very small babies experiencing higher mortality rates (NMR: 0.48 vs. 0.21; IMR: 0.64 vs. 0.34; UMR: 0.76 vs. 0.44). The mortality rates in cases where the child’s birth weight was not known were much higher. The rates of neonatal mortality were higher for babies delivered by Caesarean section (NMR: 0.37 vs. 0.25); the estimated infant and under-five rates for Caesarean babies were also higher but within confidence intervals of the non-Caesarean delivery estimates. Women who did not receive antenatal care experienced much higher mortality rates on all three measures (NMR: 0.49 vs. 0.17; IMR: 0.66 vs. 0.26; UMR: 0.74 vs. 0.31) even though the small number of women in this category made the confidence intervals much larger. Women whose survey responses indicated sexual autonomy experienced lower child mortality rates on all three measures (NMR: 0.25 vs. 0.31; IMR: 0.38 vs. 0.46; UMR: 0.48 vs. 0.57). Relatedly, women who reported IPV showed higher rates of child mortality, although only the under-five rate was significantly higher (0.54 vs. 0.46). Women who used contraceptives experienced lower child mortality rates on all three measures (NMR: 0.21 vs. 0.30; IMR: 0.30 vs. 0.46; UMR: 0.37 vs. 0.58).

**Table 4:** Neonatal, infant and under-five mortality rates in East Africa (Per 1000 Births), by demographic characteristics of mothers.

Variable	East Africa (Kenya, Uganda, Tanzania, Rwanda, Burundi)		
	NMR (95% CI)	IMR (95% CI)	UMR (95% CI)
**Residential Area**			
Rural	0.30 (0.26–0.34)	0.42 (0.37–0.47)	0.50 (0.45–0.56)
Urban	0.25 (023–0.26)	0.38 (0.36–0.40)	0.49 (0.47–0.51)
**Wealth Index**			
Poor	0.25 (0.23–0.27)	0.40 (0.37–0.43)	0.53 (0.50–0.56)
Middle	0.25 (0.22–0.28)	0.37 (0.33–0.41)	0.46 (0.42–0.50)
Rich	0.28 (0.25–0.31)	0.39 (0.36–0.43)	0.47 (0.43–0.51)
**Mother’s Age**			
<20 years	0.38 (0.31–0.47)	0.49 (0.41–0.59)	0.56 (0.47–0.66)
20–29 years	0.25 (0.23–0.27)	0.38 (0.36–0.41)	0.49 (0.46–0.52)
30–49 years	0.28 (0.25–0.31)	0.39 (0.37–0.42)	0.49 (0.46–0.53)
**Mother’s Age at First Birth**			
<20 years	0.25 (0.23–0.27)	0.39 (0.37–0.42)	0.51 (0.48–0.54)
20–29 years	0.26 (0.24–0.29)	0.39 (0.36–0.42)	0.47 (0.44–0.50)
30–49 years	0.32 (0.18–0.55)	0.47 (0.31–0.71)	0.55 (0.38–0.79)
**Husband/Partner’s Age**			
<29 years	0.29 (0.25–0.33)	0.41 (0.37–0.46)	0.53 (0.48–0.58)
30–39 years	0.23 (0.21–0.26)	0.37 (0.34–0.40)	0.46 (0.42–0.50)
40–49 years	0.28 (0.24–0.32)	0.40 (0.36–0.45)	0.50 (0.45–0.55)
50 plus years	0.29 (0.23–0.37)	0.43 (0.35–0.52)	0.60 (0.51–0.70)
**Mother’s Religion**			
Roman Catholic	0.27 (0.24–0.30)	0.42 (0.38–0.45)	0.52 (0.48–0.57)
Protestant	0.24 (0.22–0.27)	0.37 (0.34–0.40)	0.46 (0.43–0.49)
Muslim	0.29 (0.24–0.36)	0.40 (0.34–0.48)	0.49 (0.42–0.57)
No religion	0.24 (0.19–0.30)	0.38 (0.32–0.45)	0.51 (0.44–0.59)
**Mother’s Education Level**			
No education	0.25 (0.22–0.28)	0.41 (0.38–0.46)	0.57 (0.52–0.62)
Primary	0.26 (0.25–0.28)	0.40 (0.38–0.43)	0.50 (0.48–0.53)
Secondary	0.25 (0.21–0.29)	0.35 (0.30–0.40)	0.40 (0.35–0.46)
Higher	0.28 (0.18–0.45)	0.35 (0.24–0.52)	0.37 (0.25–0.53)
**Mother’s Working Status**			
No	0.26 (0.23–0.31)	0.38 (0.34–0.43)	0.48 (0.43–0.53)
Yes	0.26 (0.25–0.28)	0.40 (0.38–0.43)	0.51 (0.48–0.54)
**Father’s Working Status**			
No	0.34 (0.23–0.50)	0.49 (0.36–0.67)	0.65 (0.50–0.83)
Yes	0.26 (0.24–0.28)	0.39 (0.37–0.41)	0.49 (0.47–0.52)
**Mother’s Mass Media Usage**			
Yes	0.25 (0.22–0.28)	0.39 (0.36–0.43)	0.52 (0.48–0.56)
No	0.26 (0.25–0.28)	0.39 (0.37–0.42)	0.48 (0.46–0.51)
**Desire for Pregnancy**			
Then	0.28 (0.25–0.30)	0.42 (0.39–0.45)	0.53 (0.55–0.60)
Later	0.23 (0.20–0.26)	0.35 (0.31–0.38)	0.44 (0.40–0.49)
No more	0.27 (0.22–0.34)	0.38 (0.32–0.46)	0.45 (0.38–0.53)
**Mother’s BMI**			
<18.5	0.17 (0.13–0.23)	0.31 (0.24–0.39)	0.41 (0.34–0.50)
> = 18.5	0.28 (20.6–0.31)	0.41 (0.39–0.44)	0.52 (0.49–0.55)
**Sex of Child**			
Male	0.29 (0.27–0.31)	0.43 (0.40–0.46)	0.53 (0.51–0.57)
Female	0.23 (0.21–0.25)	0.35 (0.33–0.38)	0.45 (0.42–0.48)
**Size of Child at Birth**			
Average or larger	0.21 (0.19–0.23)	0.34 (0.32–0.36)	0.44 (0.42–0.46)
Small or very small	0.48 (0.42–0.53)	0.64 (0.58–0.71)	0.76 (0.70–0.83)
Don’t know	1.22 (0.93–1.59)	1.31 (1.01–1.68)	1.46 (1.16–1.82)
**Birth Weight (g)**			
<2500	0.65 (0.55–0.75)	0.89 (0.77–1.01)	1.02 (0.90–1.16)
2500–3500	0.15 (0.14–0.17)	0.27 (0.25–0.30)	0.36 (0.34–0.39)
>3500	0.19 (0.16–0.23)	0.31 (0.27–0.36)	0.42 (0.37–0.47)
Not weighed	0.38 (0.34–0.42)	0.51 (0.47–0.56)	0.63 (0.59–0.68)
Don’t know	1.63(1.28–2.05)	1.78(1.42–2.21)	1.93(1.55–2.36)
**Birth Order and Interval**			
2^nd^/3^rd^ child, >2 years	0.20 (0.17–0.22)	0.32 (0.29–0.35)	0.41 (0.38–0.45)
1^st^ child	0.33 (0.29–0.37)	0.44 (0.40–0.49)	0.54 (0.50–0.59)
2^nd^/3^rd^ child, ≤2 years	0.26 (0.21–0.31)	0.40 (0.34–0.46)	0.52 (0.46–0.59)
4^th^/higher child, >2 years	0.23 (0.21–0.26)	0.36 (0.32–0.39)	0.45 (0.42–0.49)
4^th^/higher child, ≤2 years	0.37 (0.32–0.43)	0.60 (0.53–0.68)	0.73 (0.65–0.81)
**Delivery by Caesarean Section**			
No	0.25 (0.24–0.27)	0.38 (0.37–0.41)	0.49 (0.47–0.51)
Yes	0.37 (0.29–0.47)	0.48 (0.39–0.59)	0.57 (0.48–0.68)
**Delivery Assistance**			
Non-health professional	0.27 (0.24–0.30)	0.40 (0.37–0.44)	0.51 (0.47–0.56)
Health professional	0.25 (0.23–0.27)	0.37 (0.35–0.40)	0.47 (0.44–0.50)
**Place of Delivery**			
Home delivery	0.26 (0.23–0.29)	0.40 (0.37–0.44)	0.52 (0.48–0.56)
Hospital/other	0.26 (0.24–0.28)	0.38 (0.36–0.41)	0.48 (0.45–0.50)
**Antenatal Care**			
No	0.49 (0.36–0.66)	0.66 (0.51–0.85)	0.74 (0.58–0.93)
Yes	0.17 (0.15–0.18)	0.26 (0.24–0.27)	0.31 (0.29–0.33)
**Sexual Autonomy**			
No	0.31 (0.27–0.36)	0.46 (0.41–0.51)	0.57 (0.51–0.62)
Yes	0.25 (0.23–0.27)	0.38 (0.35–0.40)	0.48 (0.45–0.55)
**Mother Experienced IPV**			
No	0.24 (0.22–0.27)	0.37 (0.34–0.40)	0.46 (0.43–0.50)
Yes	0.29 (0.26–0.31)	0.42 (0.39–4.5)	0.54 (0.51–0.57)
**Contraceptive Use**			
Not using	0.30 (0.28–0.32)	0.46 (0.43–0.48)	0.58 (0.56–0.62)
Using	0.21 (0.18–0.23)	0.30 (0.28–0.33)	0.37 (0.34–0.40)
**Mother Giving Sexual Favors**			
No	0.26 (0.21–0.32)	0.39 (0.33–0.46)	0.46 (0.40–0.53)
Yes	0.32 (0.13–0.75)	0.53 (0.21–1.29)	0.56 (0.23–0.73)

#### Univariate analysis

Table 5 presents a series of univariate survival analyses examining the association between various sociodemographic and behavioral characteristics and the hazard rates for neonatal, infant, and under-five mortality. This section highlights some of the statistically significant univariate predictors of mortality. Children under the age of five born to mothers in the middle (UHR = 0.83, 95% CI: 0.73–0.94, *p* = 0.005) or highest (UHR = 0.80, 95% CI: 0.70–0.90, *p*<0.001) categories of the wealth index were significantly less likely to die than those in the poorest households. Mothers aged between 20 and 29 years at first birth had a significantly lower rate of under-five mortality (UHR = 0.90, 95% CI: 0.81–1.00, *p* = 0.044) than younger mothers. Compared with young fathers, children born to fathers aged between 30 and 39 years had a significantly lower risk of neonatal and under-five mortality (NHR = 0.78, 95% CI: 0.65–0.95, *p* = 0.011; UHR = 0.83, 95% CI: 0.73–0.96, *p* = 0.010). Mothers who had completed primary (IHR = 0.85, 95% CI: 0.75–0.97, *p* = 0.012; UHR = 0.80, 95% CI: 0.72–0.90, *p*<0.001) or secondary (IHR = 0.79, 95% CI: 0.65–0.97, *p* = 0.023; UHR = 0.69, 95% CI: 0.57–0.82, *p*<0.001) levels of education had a significantly lower rate of infant and under-five mortality than those with no education. Mothers with a BMI greater than or equal to 18.5 kg/m^2^ bore children with a significantly higher risk of mortality compared to mothers with a BMI lower than 18.5 kg/m^2^ (NHR = 1.59, 95% CI: 1.09–2.32, *p* = 0.015; IHR = 1.40, 95% CI: 1.05–1.87, *p* = 0.021; UHR = 1.30, 95% CI: 1.01–1.66, *p* = 0.044). Female children were significantly less likely to die compared to males (NHR = 0.77, 95% CI: 0.67–0.89, *p*<0.001; IHR = 0.81, 95% CI: 0.72–0.91, p<0.001; UHR = 0.82, 95% CI: 0.75–0.91, *p*<0.001).

**Table 5:** Univariate analysis of the association between neonatal, infant, and under-five mortality in East Africa and sociodemographic and behavioral characteristics.

Variable	Neonatal Mortality Hazard Rate			Infant Mortality Hazard Rate			Under-five Mortality Hazard Rate		
	NHR	95% CI	*P*	IHR	95% CI	*p*	UHR	95% CI	*p*
**Residential Area**									
Rural	1.00			1.00			1.00		
Urban	0.97	0.77–1.21	0.768	1.00	0.84–1.20	0.997	1.12	0.96–1.32	0.159
**Wealth Index**									
Poor	1.00			1.00			1.00		
Middle	1.01	0.85–1.21	0.905	0.89	0.77–1.03	0.113	0.83	0.73–0.94	0.005**
Rich	1.04	0.88–1.24	0.619	0.89	0.77–1.02	0.091	0.80	0.70–0.90	<0.001**
**Mother’s Age**									
<20 years	1.00			1.00			1.00		
20–29 years	0.58	0.44–0.76	<0.001**	0.62	0.49–0.79	<0.001**	0.66	0.53–0.83	<0.001**
30–49 years	0.60	0.46–0.79	<0.001**	0.64	0.51–0.81	<0.001**	0.66	0.53–0.82	<0.001**
**Mother’s Age at First Birth**									
<20 years	1.00			1.00			1.00		
20–29 years	0.96	0.83–1.10	0.52	0.94	0.84–1.06	0.320	0.90	0.81–1.00	0.044**
30–49 years	1.31	0.67–2.55	0.43	1.30	0.78–2.16	0.317	1.19	0.75–1.88	0.456
**Husband/Partner’s Age**									
<29 years	1.00			1.00			1.00		
30–39 years	0.78	0.65–0.95	0.011**	0.88	0.75–1.04	0.133	0.83	0.73–0.96	0.010**
40–49 years	0.90	0.72–1.11	0.321	0.89	0.75–1.07	0.226	0.84	0.72–0.99	0.037**
50 plus years	1.04	0.78–1.39	0.790	1.08	0.85–1.36	0.525	1.11	0.91–1.36	0.295
**Mother’s Religion**									
Roman Catholic	1.00			1.00			1.00		
Protestant	0.92	0.79–1.08	0.322	0.89	0.78–1.02	0.083	0.87	0.77–0.98	0.025**
Muslim	1.11	0.88–1.41	0.374	1.00	0.81–1.24	0.989	0.97	0.80–1.17	0.743
No religion	0.87	0.66–1.13	0.293	0.91	0.73–1.12	0.361	0.98	0.81–1.17	0.782
**Mother’s Education Level**									
No education	1.00			1.00			1.00		
Primary	0.94	0.80–1.10	0.435	0.85	0.75–0.97	0.012**	0.80	0.72–0.90	<0.001**
Secondary	0.98	0.77–1.25	0.880	0.79	0.65–0.97	0.023**	0.69	0.57–0.82	<0.001**
Higher	0.83	0.44–1.57	0.565	0.62	0.37–1.06	0.078	0.48	0.29–0.81	0.005**
**Mother’s Working Status**									
No	1.00			1.00			1.00		
Yes	1.01	0.83–1.22	0.920	1.05	0.90–1.23	0.552	1.05	0.91–1.21	0.490
**Father’s Working Status**									
No	1.00			1.00			1.00		
Yes	0.83	0.55–1.25	0.378	0.86	0.62–1.19	0.352	0.81	0.61–1.07	0.139
**Mother’s Mass Media Usage**									
Yes	1.00			1.00			1.00		
No	0.99	0.86–1.14	0.870	0.92	0.82–1.04	0.176	0.86	0.78–0.96	0.006
**Desire for Pregnancy**									
Then	1.00			1.00			1.00		
Later	0.82	0.70–0.97	0.023**	0.84	0.73–0.97	0.014**	0.84	0.75–0.95	0.005**
No more	1.04	0.81–1.34	0.741	0.96	0.79–1.18	0.714	0.90	0.75–1.08	0.249
**Mother’s BMI**									
<18.5	1.00			1.00			1.00		
> = 18.5	1.59	1.09–2.32	0.015**	1.40	1.05–1.87	0.021**	1.30	1.01–1.66	0.044**
**Sex of Child**									
Male	1.00			1.00			1.00		
Female	0.77	0.67–0.89	<0.001**	0.81	0.72–0.91	<0.001**	0.82	0.75–0.91	<0.001**
**Size of Child at Birth**									
Average or larger	1.00			1.00			1.00		
Small or very small	2.05	1.75–2.39	<0.001**	1.82	1.61–2.06	<0.001**	1.69	1.51–1.88	<0.001**
Don’t know	6.42	4.74–8.70	<0.001**	4.53	3.39–6.06	<0.001**	3.86	2.96–5.04	<0.001**
**Birth Weight (Kg)**									
<2500	1.00			1.00			1.00		
2500–3500	0.24	0.20–0.30	<0.001**	0.31	0.26–0.37	<0.001**	0.36	0.31–0.42	<0.001**
>3500	0.30	0.22–0.40	<0.001**	0.36	0.28–0.45	<0.001**	0.41	0.33–0.50	<0.001**
Not weighed	0.73	0.60–0.90	0.003**	0.69	0.58–0.83	<0.001**	0.71	0.60–0.84	<0.001**
Don’t know	2.71	1.95–3.77	<0.001**	2.23	1.64–3.04	<0.001**	2.09	1.55–2.82	<0.001**
**Birth Order and Interval**									
2^nd^/3^rd^ child, >2 years	1.00			1.00			1.00		
1^st^ child	1.68	1.39–2.01	<0.001**	1.38	1.18–1.61	<0.001**	1.29	1.12–1.49	<0.001**
2^nd^/3^rd^ child, ≤2 years	1.45	1.12–1.97	0.004	1.26	1.02–1.55	0.034**	1.25	1.04–1.50	0.016**
4^th^/higher child, >2 years	1.19	0.97–1.46	0.088	1.08	0.92–1.26	0.361	1.07	0.93–1.23	0.336
4^th^/higher child, ≤2 years	2.06	1.63–2.59	<0.001**	2.01	1.66–2.45	<0.001**	1.83	1.53–2.17	<0.001**
**Delivery by Caesarean Section**									
No	1.00			1.00			1.00		
Yes	1.34	1.00–1.78	0.047**	1.09	0.85–1.39	0.509	1.09	0.88–1.36	0.441
**Delivery Assistance**									
Non-health professional	1.00			1.00			1.00		
Health professional	0.89	0.72–1.10	0.276	0.93	0.79–1.20	0.383	0.91	0.79–1.05	0.205
**Place of Delivery**									
Home delivery	1.00			1.00			1.00		
Hospital/other	0.79	0.67–0.93	0.005**	0.81	0.71–0.93	0.003**	0.83	0.74–0.93	0.001**
**Antenatal Care**									
No	1.00			1.00			1.00		
Yes	0.33	0.23–0.49	<0.001**	0.34	0.24–0.47	<0.001**	0.36	0.26–0.48	<0.001**
**Sexual Autonomy**									
No	1.00			1.00			1.00		
Yes	0.80	0.68–0.94	0.006	0.82	0.72–0.93	0.003**	0.84	0.75–0.94	0.002**
**Contraceptive Use**									
Not using	1.00			1.00			1.00		
Using	0.69	0.59–0.81	<0.001**	0.62	0.55–0.71	<0.001**	0.58	0.52–0.65	<0.001**
**Mother Giving Sexual Favors**									
No	1.00			1.00			1.00		
Yes	1.21	0.49–2.99	0.678	1.34	0.52–3.49	0.545	1.23	0.50–3.05	0.656

Two asterisks (**) denotes the *p*-value is <0.001, and one asterisk (*) denotes the *p*-value is <0.05.

Children whose mothers perceived them as small or very small had a significantly higher risk of mortality than those of average or larger size (NHR = 2.05, 95% CI: 1.75–2.39, *p*<0.001; IHR = 1.82, 95% CI: 1.61–2.06, *p*<0.001; UHR = 1.69, 95% CI: 1.51–1.88, *p*<0.001). Furthermore, there was a significantly lower risk of mortality for children who weighed between 2500 and 3500 grams (NHR = 0.24, 95% CI: 0.20–0.30, *p*<0.001; IHR = 0.31, 95% CI: 0.26–0.37, *p*<0.001; UHR = 0.36, 95% CI: 0.31–0.42, *p*<0.001) and above 3500 grams (NHR = 0.30, 95% CI: 0.22–0.40, *p*<0.001; IHR = 0.36, 95% CI: 0.28–0.45, *p*<0.001; UHR = 0.41, 95% CI: 0.33–0.50, *p*<0.001) compared with those who weighed below 2500 grams. Babies delivered by Caesarean section had a significantly higher risk of neonatal death compared with non-Caesarean delivery (NHR = 1.34, 95% CI: 1.00–1.78, *p* = 0.047). However, children born at hospitals had a significantly lower risk of mortality than those born at home (NHR = 0.79, 95% CI: 0.67–0.93, *p* = 0.005; IHR = 0.81, 95% CI: 0.71–0.93, *p* = 0.003; UHR = 0.83, 95% CI: 0.74–0.93, *p* = 0.001). Mothers who received antenatal care gave birth to children with a significantly lower risk of mortality compared with those who did not receive care (NHR = 0.33, 95% CI: 0.23–0.49, *p*<0.001; IHR = 0.34, 95% CI: 0.24–0.47, *p*<0.001; UHR = 0.36, 95% CI: 0.26–0.48, *p*<0.001). Mothers who were able to assert sexual autonomy bore children with a significantly lower risk of infant and under-five mortality than those who did not (IHR = 0.82, 95% CI: 0.72–0.93, *p* = 0.003; UHR = 0.84, 95% CI: 0.75–0.94, *p* = 0.002). There was a significantly lower risk of mortality among children born to mothers who use contraceptives relative to those who did not (NHR = 0.69, 95% CI: 0.59–0.81, *p*<0.001; IHR = 0.62, 95% CI: 0.55–0.71, *p*<0.001; UHR = 0.58, 95% CI: 0.52–0.65, *p*<0.001).

Table 6 details the results of a multivariate analysis examining the relative effects of selected demographic and behavioral characteristics on the three child mortality rates. The predictor variables included in each model are those found significantly associated with the particular measure of mortality in the univariate analysis (Table 5), using a stepwise backwards elimination procedure and removing any predictor variables that produced multi-collinearity. This section discusses the model for each mortality outcome in turn.

**Table 6:** Multivariate analysis of the association between neonatal, infant, and under-five mortality in East Africa and sociodemographic and behavioral characteristics.

Variable	Neonatal Mortality Hazard Rate			Infant Mortality Hazard Rate			Under-five Mortality Hazard Rate		
	NHR	95% CI	*P*	IHR	95% CI	*p*	UHR	95% CI	*p*
**Wealth Index**									
Poor	1.00								
Middle	1.19	0.66–2.13	0.565						
Rich	0.62	0.30–1.29	0.199						
**Husband/Partner’s Age**									
<29 years				1.00			1.00		
30–39 years				0.88	0.63–1.24	0.470	0.85	0.63–1.14	0.278
40–49 years				1.02	0.68–1.53	0.918	0.85	0.58–1.23	0.375
50 plus years				1.02	0.61–1.69	0.939	0.96	0.62–1.51	0.871
**Mother’s Working Status**									
No	1.00			1.00			1.00		
Yes	1.77	1.10–2.85	0.019**	1.46	1.06–1.99	0.020**	1.48	1.11–1.96	0.007
**Mother’s Mass Media Usage**									
Yes	1.00			1.00			1.00		
No	2.80	1.39–5.63	0.004**	1.16	0.89–1.51	0.268	1.05	0.83–1.32	0.696
**Mother’s BMI**									
<18.5	1.00			1.00			1.00		
> = 18.5	2.67	1.14–6.23	0.023**	2.13	1.32–3.44	0.002**	1.74	1.16–2.61	0.007
**Sex of Child**									
Male	1.00								
Female	0.68	0.43–1.08	0.100						
**Size of Child at Birth**									
Average or larger	1.00			1.00			1.00		
Small or very small	1.96	1.20–3.21	0.007**	1.26	0.91–1.73	0.163	1.23	0.92–1.65	0.166
Don’t know	3.41	1.48–7.86	0.004**	1.97	0.95–4.11	0.070	1.75	0.80–3.84	0.164
**Birth Weight (g)**									
<2500	1.00			1.00			1.00		
2500–3500	0.78	0.32–1.90	0.584	0.25	0.16–0.40	<0.001**	0.28	0.19–0.43	<0.001**
>3500	0.75	0.23–2.43	0.629	0.32	0.18–0.55	<0.001**	0.33	0.20–0.55	<0.001**
Not weighed	2.05	0.61–6.86	0.246	0.64	0.39–1.04	0.073	0.58	0.37–0.91	0.017**
Don’t know	11.5	3.59–36.80	<0.001**	3.12	1.68–5.81	<0.001**	2.63	1.40–4.94	0.003**
**Birth Order and Interval**									
2^nd^/3^rd^ child, >2 years				1.00			1.00		
1^st^ child				1.32	0.88–1.99	0.186	1.27	0.88–1.84	0.201
2^nd^/3^rd^ child, ≤2 years				0.92	0.54–1.54	0.741	1.05	0.66–1.68	0.837
4^th^/higher child, >2 years				1.12	0.78–1.59	0.537	1.12	0.82–1.55	0.480
4^th^/higher child, ≤2 years				1.53	0.94–2.48	0.089	1.65	1.08–2.52	0.022**
**Place of Delivery**									
Home delivery	1.00			1.00			1.00		
Hospital/other	1.99	0.77–5.15	0.159	1.34	0.90–2.00	0.147	1.21	0.85–1.74	0.294
**Antenatal Care**									
No	1.00			1.00			1.00		
Yes	0.32	0.17–0.62	<0.001**	0.53	0.31–0.92	0.023**	0.54	0.32–0.91	0.020**
**Contraceptive Use**									
Not using	1.00			1.00			1.00		
Using	0.37	0.22–0.63	<0.001**	0.40	0.30–0.54	<0.001**	0.36	0.27–0.47	<0.001**
**Mother Giving Sexual Favors**									
No	1.00								
Yes	0.64	0.19–2.15	0.468						
**Sexual Autonomy**									
No	1.00			1.00			1.00		
Yes	0.80	0.68–0.94	0.006**	0.82	0.72–0.93	0.003**	0.84	0.75–0.94	0.002**

Two asterisks (**) denotes the *p*-value is <0.001, and one asterisk (*) denotes the *p*-value is <0.05.

In the model for neonatal mortality, women who exercised sexual autonomy displayed a significantly lower risk of neonatal death than women who did not (NHR = 0.80, 95% CI: 0.68–0.94, *p* = 0.006), net of the other predictor variables in the model. By contrast, working mothers (NHR = 1.77, 95% CI: 1.10–2.85, *p* = 0.019), those who made use of mass media (NHR = 2.80, 95% CI: 1.39–5.63, *p* = 0.004), and those with a BMI of 18.5 or higher (NHR = 2.67, 95% CI: 1.14–6.23, *p* = 0.023) all suffered neonatal loss at significantly higher rates than their counterparts on each predictor characteristic. For both of the variables related to the child’s birth weight, in cases where the weight was not known, the risk of neonatal death was significantly higher. With respect to the mother’s perception of birth size, babies of unknown weight were more likely to die than those perceived to be of average or of larger size (NHR = 3.41, 95% CI: 1.48–7.86, *p* = 0.004). When their actual birth weight was not known, neonatal death rates were significantly higher rates than that of the smallest babies—those under 2500 grams (NHR = 11.5, 95% CI: 3.59–36.80, *p*<0.001).

Mothers who received antenatal care (NHR = 0.32, 95% CI: 0.17–0.62, *p*<0.001) and those who used contraceptives (NHR = 0.37, 95% CI: 0.22–0.63, *p*<0.001) had a significantly lower risk of neonatal mortality.

The model for infant mortality shows a similar, but not identical, set of significant predictor values. Women exerting sexual autonomy experienced lower infant mortality (IHR = 0.82, 95% CI: 0.72–0.93, *p* = 0.003), while working mothers (IHR = 1.46, 95% CI: 1.06–1.99, *p* = 0.020) and those with a BMI of 18.5 or higher (IHR = 2.13, 95% CI: 1.32–3.44, *p* = 0.002) suffered higher infant mortality. Perceived birth weight did not emerge as a significant predictor, but actual birthweight did; babies who weighed 2500–3500 grams at birth (IHR = 0.25, 95% CI: 0.16–0.40, *p*<0.001) and those weighing greater than 3500 grams (IHR = 0.32, 95% CI: 0.18–0.55, *p*<0.001) were less likely to die as infants than those who weighed under 2500 grams at birth. However, when the baby’s weight was not known, the risk of infant death was much higher than for the smallest babies (IHR = 3.12, 95% CI: 0.18–0.55, *p*<0.001). As with neonatal mortality, mothers who received antenatal care (IHR = 0.53, 95% CI: 0.31–0.92, *p* = 0.023) and those who used contraceptives (IHR = 0.40, 95% CI: 0.30–0.54, *p*<0.001) each had a significantly lower risk of infant mortality.

The model for under-five child mortality shows a smaller set of significant predictors. Women’s sexual autonomy was significant in lowering the risk of child mortality (UHR = 0.84, 95% CI: 0.75–0.94, *p* = 0.002). Mother’s work status and BMI did not have a significant effect. The child’s birthweight did serve as a significant predictor, with varying effects depending on the weight result. Babies weighing 2500–3500 grams at birth (UHR = 0.28, 95% CI: 0.19–0.43, *p*<0.001), those greater than 3500 grams (UHR = 0.33, 95% CI: 0.20–0.55, *p*<0.001), and those who were not weighed (UHR = 0.58, 95% CI: 0.37–0.91, p = 0.017) were less likely to die before age five than those who weighed under 2500 grams at birth. When the infants’ weight was not known, risk of death before age five was much higher than for the smallest of babies (UHR = 2.63, 95% CI: 1.40–4.94, *p* = 0.003).

As was true for both neonatal and infant mortality, mothers who received antenatal care (UHR = 0.54, 95% CI: 0.32–0.91, *p* = 0.020) and those who used contraceptives (UHR = 0.36, 95% CI: 0.27–0.47, p<0.001), each had a significantly lower risk of losing their children before age five, net of all the other factors in the model.

## Discussion

Autonomous decision-making encompasses independence, self-determination, and freedom from external coercion, which is key when engaging in sexual activity [13]. Compared to younger mothers (*p*<0.05) findings indicate a significant decrease in the risk of mortality for children born to mothers aged between 20–29 and 30–49 years of age. According to the World Health Organization, infant mortality rates among children born to younger mothers were 73% higher compared to older mothers [14]. Immediate pressure is often placed on girls who marry early to prove fertility because their partner has paid a dowry [15]. Adolescent mothers have a higher risk (35–55%) of delivering infants who are pre-term and of low birth weight compared to women aged 20 and older [15]. In Tanzania, mortality rates among women under 20 years is close to twice as high (164 per 1000) as women 20 years of age and older (64 per 1000). Increased mortality rates can be attributed in part to inadequate prenatal and postpartum healthcare, untreated sexually transmitted diseases, and reproductive immaturity [15].

The study further revealed that women who experienced intimate partner violence (IPV) gave birth to children who had higher mortality rates (*p*<0.05) compared to those who did not experience IPV. Meta-analysis of articles from Medline ascertains the impact of IPV on woman’s reproductive health and pregnancy outcomes. Data suggests that IPV reduces sexual autonomy and increases the risk of unintended pregnancies and multiple abortions [16]. Findings of this study concur with our results which showed that violence against women significantly increased the risk for low birth weight infants, preterm delivery and neonatal death. Correspondingly, a study in Nicaragua analyzed the effects of physical and sexual violence on infant mortality. Results indicated that the risk of death in infancy or before five years of age was more than six times greater if a mother was exposed to both physical and sexual violence by a current or former partner at any point in time, establishing a clear causal link between violence and mortality [17]. In fact, 27% of fatalities of children under the age of five can be attributed to physical or sexual violence committed by a partner [17]. Subsequently, there is a need to empower vulnerable adolescents who are victims of early marriages and pregnancies to disclose IPV and receive treatment for both physical and emotional complaints and concerns over the use of contraception.

Enhancement of efficacy for sexual self-protective behavior is greater when young women are more advanced in cognitive autonomy [18]. A greater capacity to communicate openly among young women and their partners leads to better decision-making skills with regards to sexual behavior. Findings of this study indicated that children born to women who consent to engage in various sexual activities had significantly lower mortality rates (*p*<0.05) than those who did not give consent.

Results indicated that children born to mothers who practiced sexual autonomy and used contraceptives had a significantly lower risk of mortality than those who did not practice sexual autonomy and did not use contraceptives. The risks of pregnancy and infant death were reduced by a pooled average of 6.8% to 2.6% for each month of contraceptive use [19]. Contraceptive use can impact infant mortality by 1) preventing unwanted/unplanned pregnancies (if born, some may die prematurely) and by 2) delaying future births and decreasing birthing intervals, thus lowering the fertility rates [19]. By lengthening birth intervals, contraceptive use can improve maternal nutrition leading to enhanced nourishment of infants and improved likelihood of survival. The ability to make adequate and informed decisions on contraceptive use occurs as a result of one’s level of sexual autonomy.

For fourth or higher birth order children with short birth intervals born to mothers who practiced sexual autonomy, the risk of under-five mortality significantly increased compared with second or third birth order children with a longer birth interval born to mothers who did not practice sexual autonomy. Similarly, there was a significant increase in the risk of mortality for second or third birth order children with a short birth interval and fourth or higher birth order children with a short birth interval compared to second or third birth order children with a longer birth interval. An analysis of DHS data from Nigeria on determinants of neonatal mortality in Nigeria exhibited similar findings to our study. From the DHS data it was shown that children of birth order (2–4) born with an interval of less than two years were at a higher risk of dying than those with birth intervals longer than two years [11]. When we sought to find out whether the period before becoming pregnant influenced mortality, the data concluded that children born to mothers who desired to wait before becoming pregnant had a significantly lower risk of mortality. Deaths are attributed to short-interval births and the effect back-to-back pregnancies has on maternal well-being combined with inadequate healthcare and financial resources to care for the children [11]. This can be prevented if partners plan for pregnancy together. This can happen effectively by encouraging female empowerment and acknowledging and accepting the practice of sexual autonomy.

Exchange of sex for money or economic gain is sometimes disguised though the influence is clear; women who lack their own independent means of financial support felt less able or less willing to refuse their husband’s sexual advances compared to women with access to financial resources [20]. As much as this may be overlooked, the fact that women are less likely to refuse advances has implications on future pregnancy outcomes and their offspring. Results from our study determined that mothers who give sexual favors for economic gain gave birth to children with higher mortality rates than those who did not perform sexual favors. A literature review of 45 qualitative and quantitative studies focusing on the extent of age mixing and economic transactions in sexual relationships of adolescent girls in sub-Saharan Africa indicated several central motivations of adolescents other than monetary benefits. The evidence shows that girls have limited negotiating power, less control of sexual practices within partnerships like condom use, and are predisposed to partner violence [21]. The grouping of factors that reduce sexual autonomy engenders negative reproductive consequences among adolescents which contribute to the increased risk of neonatal and infant mortality.

There are some limitations to this study that should be taken into consideration. We only included five countries in the analysis that represent the East African community. We also did not make any country comparisons as our objective was not to assess the similarities or differences between the countries. The use of DHS data for this secondary analysis limited our ability to assess the validity of women’s self-report on sexual autonomy; however, other studies have found similar reports on women’s independence using DHS data [22–25]. This data was also collected cross-sectionally; we are therefore unable to fully explain what the mechanism is between sexual autonomy as a protective factor for all three reported mortalities in which our study proves a strong association. Longitudinal studies will be especially important in determining directionality of the observed associations. Despite these limitations, the findings of this study contribute to our understanding of sexual autonomy and its place in ensuring positive health outcomes for the child. Additional studies are needed to further explore the strong association between sexual autonomy and neonatal, infant, and child survival among different age groups. Qualitative analysis is also needed in exploring sexual relationships, women’s empowerment, and health outcomes. Sexual autonomy has commonly been overlooked in the literature as was evident from our literature review; yet it is evident from our study that a strong association exists. Other studies should also examine whether women who report sexual autonomy have better relationships. This study provides an avenue for better understanding on how women’s autonomy and empowerment can improve health outcomes.

## Conclusion

Sexual autonomy is of paramount importance for women across the world–specifically for women in Eastern Africa who are at increased risk for infant, child, and neonatal mortality. Various factors contribute to mortality of offspring, including culture, education, religion and wealth in addition to maternal, paternal, neonatal, and health system factors—all of which determine if survival or death will occur. We argue that increasing education of women, preventing pregnancy until 20 years of age or older, and allowing women sexual autonomy all contribute to increased survival of offspring. Women should be informed, empowered, and autonomous concerning their reproductive health. The research presented in this manuscript is important because empowering women and supporting gender equality are an area of focus that is also expressed in the Sustainable Development Goals (SDGs). A better understanding of the situations where autonomy is associated with improved health outcomes can assist policymakers in planning and prioritizing their investments.

### Ethics approval and consent to participate

The study used secondary data from Measure DHS and does not require ethical approval.
